# Endoscopic Assessment and Management of Colorectal Serrated Lesions: Current Comparative Practice, Challenges, and Future Directions

**DOI:** 10.1111/den.70197

**Published:** 2026-06-10

**Authors:** Toshio Uraoka, Yasushi Yamasaki, Kenichiro Imai, Hirohito Tanaka, Keigo Sato, Yuki Itoi, Hiroko Hosaka, Shiko Kuribayashi, Hemchand Ramberan, Yoji Takeuchi

**Affiliations:** ^1^ Department of Gastroenterology and Hepatology Gunma University Graduate School of Medicine Gunma Japan; ^2^ Department of Gastroenterology and Hepatology Okayama University Graduate School of Medicine, Dentistry and Pharmaceutical Sciences Okayama Japan; ^3^ Division of Endoscopy Shizuoka Cancer Center Shizuoka Japan; ^4^ Sentara Health Gastroenterology Specialists Newport News Virginia USA

**Keywords:** Colorectal cancer, endoscopic resection, serrated lesion, sessile serrated lesion, sessile serrated lesion with dysplasia

## Abstract

Serrated‐carcinoma pathway accounts for approximately 15%–30% of sporadic colorectal cancers (CRC) and contributes disproportionately to right‐sided interval cancers. Key precursors include sessile serrated lesion (SSL) and SSL with dysplasia (SSLD); traditional serrated adenoma, and also the recently described superficially serrated adenoma which warrants attention. However, high‐quality evidence guiding best practice for resection indications, technique selection, and post‐resection surveillance intervals remains limited and varied, with differences between Western and Japanese guidelines. This narrative review integrates clinicopathologic and practical perspectives to provoke further discussion for a standardized universal guideline approach. For nondysplastic SSLs, cold polypectomy techniques may be considered in selected lesions with defined margins and detailed documentation, whereas those with suspected dysplasia, non‐lifting or scarring, requiring en bloc resection, favor endoscopic mucosal resection or endoscopic submucosal dissection for accurate histopathology. Surveillance should be prioritized by histopathology and completeness of resection, with an index shorter interval assessment of the resection site at 6–12 months after piecemeal resection or when margins are involved or indeterminate, followed by risk‐tiered intervals. Because the detection of serrated lesions determines resection type, quality and surveillance interval, endoscopists should standardize bowel preparation, lesion assessment after mucus cap clearance, use image‐enhanced endoscopy when appropriate, and apply targeted dye spray when borders are indistinct. Finally, we outline priority research; prospective, serrated lesion‐focused cohorts and trials—to ascertain durable outcomes of cold snare resection strategies, refine surveillance intervals, and reconcile regional practice differences, with the overarching goal of reducing post‐colonoscopy interval CRC and overall CRC incidence with high‐quality colonoscopy.

## 1 Introduction

Although colorectal cancer (CRC) has long been known to arise through the adenoma–carcinoma sequence and de novo pathways, the serrated polyp‐carcinoma sequence (serrated pathway) has more recently been recognized as a different path to carcinogenesis with distinctive features, including enrichment of *BRAF* or *KRAS* mutations, CpG island methylation phenotype, and microsatellite instability [1, 2, 3, 4]. The serrated pathway is estimated to account for approximately 15%–30% of sporadic CRCs, representing a substantial proportion [1, 3, 4]. Large serrated lesions are particularly common in women and older adults and are considered to carry a higher risk of malignant transformation. In addition, lifestyle factors such as obesity, smoking, and alcohol consumption appear to influence the development and progression of serrated lesions on the colorectum [1, 4].

The World Health Organization (WHO) classifies serrated lesions into hyperplastic polyps (HP), traditional serrated adenomas (TSA), sessile serrated lesions (SSL), and SSL with dysplasia (SSLD) [5, 6, 7, 8]. SSL and SSLD are known to be easily missed or incompletely resected due to their ill‐defined surface features and indistinct margins, contributing to right‐sided interval CRC [4, 6, 9, 10]. Therefore, deliberate meticulous evaluation for detection and quality of endoscopic resection with appropriate technique selection are crucial to reducing the incidence and mortality of CRC through the serrated pathway as well as the adenoma–carcinoma pathway. Though endoscopic resection of colorectal serrated lesions excluding HP is also expected to reduce CRC occurrence, various opinions exist, and a unified consensus is lacking [4, 6, 8]. The present review discusses an international perspective on current practices, challenges, and future directions of endoscopic assessment and management of serrated lesions, especially SSL and SSLD in the colorectum. This invited review is intended for an international endoscopists and guideline developers' audience hoping to provide practical and universally applicable recommendations while contrasting current Japanese and Western practice.

## 2 Clinicopathological Features of Serrated Lesion

### 2.1 Sessile Serrated Lesion

The Japanese Society for Cancer of the Colon and Rectum (JSCCR) has traditionally used the term “SSA/P” in its cancer classification [11]. In contrast, the 2019 WHO classification revision unified the terminology to “SSL” and notably relaxed the histopathological diagnostic criteria when considering the structural alterations [5, 6, 7]. These include crypt base expansion, bifurcated or horizontal crypt bases, and L‐ or boot‐shaped crypts. However, the presence of serrated crypts with one or more obvious structural alterations is sufficient for an SSL diagnosis. Revision of the diagnostic criteria and expanding the definition of SSL aims to broaden the recognition of the clinical significance of these lesions and the importance of high‐quality endoscopic resection, thereby reducing the risk of CRC. Due to this revised definition, SSA/P diagnosed under JSCCR criteria is not necessarily identical to SSL as defined by WHO 2019 [5]. This distinction is important for both epidemiology and management strategies. At the molecular level, most SSL harbor *BRAF* mutations and exhibit CIMP‐high status. Some acquire MLH1 promoter methylation, progressing to SSLD and ultimately to MSI‐high CRC [1, 3, 7, 12].

**FIGURE 1:**
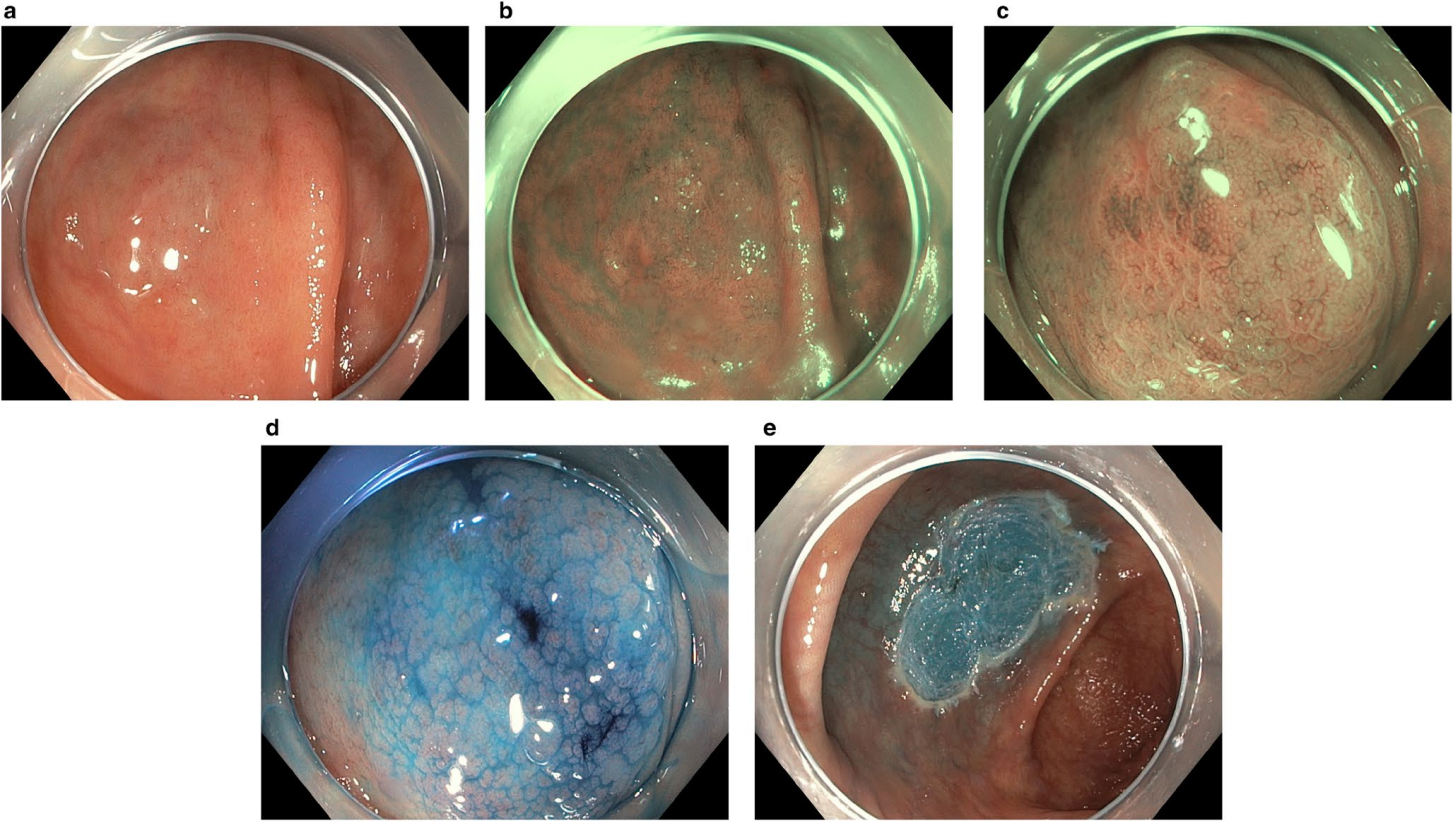
Representative example of SSL observed using a Western standard colonoscope (Olympus 190 series). These images illustrate that key endoscopic features of SSL can be recognized and adequately managed using high‐definition and near‐focus imaging modalities widely available in Western practice, even without full optical magnification. (a) White‐light imaging showing a flat, pale lesion with indistinct margins. (b) NBI demonstrating enhanced surface and vascular patterns. (c) NBI with near focus highlighting dilated superficial vessels and dark spots within crypts. (d) Chromoendoscopy with dye spray clearly delineating lesion borders. (e) Clean post‐resection base confirming complete removal and margin assessment.

SSL is typically located in the right‐side colon and appears flat or slightly elevated, pale, with a mucus cap and indistinct borders [13, 14]. With the magnifying or image‐enhanced endoscopy (IEE) (e.g., narrow band imaging [NBI] or blue laser imaging [BLI]) inspection, where available, SSL correlates to Japan NBI Expert Team (JNET) Type 1 [15] and often reveals varicose microvascular vessels and dark spots inside crypts [16, 17], which correspond to the Kudo II‐O pit pattern [18]. In regions where optical full zoom magnification is not routinely available, these endoscopic criteria can be applied using high‐definition, near‐focus function (non‐full zoom magnification) that are widely used in Western practice [19]. Representative examples using high‐definition and near‐focus imaging from Western practice are shown in Figure 1. The Workgroup serrAted polypS and Polyposis (WASP) criteria, which consists of a cloud‐like surface, indistinct margin, dark spot and irregular shape helps to distinguish SSL from HP [20]. However, it is not possible to assess certain histological features, such as abnormal dilation of glandular ducts at the base of mucosal glands by endoscopic interrogation of the lesion surface. Therefore, it is important to evaluate suspected lesions meticulously by comprehensively utilizing various endoscopic techniques to help identify characteristic morphologic features [17].

### 2.2 Sessile Serrated Lesion With Dysplasia

Two primary pathways are recognized for the progression of SSLs to CRC [1, 2, 3, 4, 7]. One is the MSI pathway, which is often associated with loss of expression due to promoter methylation of the *MLH1* gene (CIMP pathway). The other is the MSS pathway, in which *TP53* gene mutations may be involved. SSLD refers to the change of cytologic dysplasia within SSL. Its histology is not uniform and encompasses multiple architectural and cytologic patterns, including serrated‐type and conventional intestinal‐type dysplasia [12, 17, 21].

When specific endoscopic features such as a two‐step elevation, pedunculation, nodule, central depression, or focal erythema are observed on SSL's surface, the coexistence of dysplasia or carcinoma should be suspected [21, 22, 23]. A high predictive probability for accurate diagnosis is possible by deliberate, high‐quality, and comprehensive interrogation of the suspected lesion. Additionally, utilizing both magnifying image‐enhanced endoscopy and pit‐pattern analysis enables improved endoscopic diagnosis of dysplasia with high histopathologic concordance. This is achieved by identifying specific high‐risk findings, such as JNET classifications Type 2A and 2B, or Kudo's pit patterns IIIs, IIIL, IV, and V according to histology or grading of tumor in the dysplasia area [21, 22, 23, 24].

### 2.3 Superficial Serrated Lesion

Superficially serrated adenoma (SuSA), recently proposed by Sekine et al., has drawn growing interest in endoscopic diagnosis [25, 26]. By histopathology, serrations are confined to the superficial crypts, while deeper portions show adenomatous change, and notably, the adenomatous component does not exhibit the elongated, spindle‐shaped nuclei typical of conventional adenoma. The proliferative zone commonly localizes to the mid‐crypt. Molecularly, SuSA frequently harbors *KRAS* mutations with RSPO3‐pathway activation, and characteristic PTPRK–RSPO3 fusions are reported in 80%–90% of cases [25, 26, 27, 28].

Because robust evidence demonstrating malignant transformation is lacking and current data are largely based on histopathologic descriptions and small case‐series observations, and the nosologic position of SuSA still requires clarification, the implications for endoscopic diagnosis and patient management currently remain uncertain.

## 3 Current Practices

Although there is no published evidence that directly demonstrates the morbidity and mortality‐reducing effects of endoscopic resection for SSLs, its availability is strongly anticipated [4, 20, 29]. This expectation stems primarily from two key factors. First, SSLs are precursor lesions that possess genetic mutations indicative of multi‐step carcinogenesis to CRC via the serrated polyp pathway, suggesting that their removal may suppress CRC incidence and cancer‐related death. Secondly, though recent accumulated evidence suggested that serrated lesions have a lower proportion of submucosal invasive cancer than conventional adenomas, research suggesting the rapid progression to deeply invasive cancer in serrated lesions with dysplasia strongly underscores the necessity for timely endoscopic resection as a crucial strategy for CRC prevention [4, 30, 31]. Therefore, endoscopic resection for (mostly benign) serrated lesions would be a preventive approach for CRC. Although the most important issue is the treatment efficacy, safety should also be considered as equally important in cancer prevention strategies. Thus, endoscopic resection techniques with lower risk might be applicable for serrated lesions.

### 3.1 Management of Serrated Lesions: Japanese Versus Western Perspectives

#### 3.1.1 Western Perspective

The Western guidelines including the European Society of Gastrointestinal Endoscopy (ESGE), the US Multi‐Society Task Force (USMSTF) generally favors a broad “resect‐all” approach to SSLs [32, 33], aiming to maximize colorectal cancer prevention and address the difficulty of reliably distinguishing SSL from HP endoscopically. The 2024 American Gastroenterological Association (AGA) document represents a Clinical Practice Update providing expert opinion–based Best Practice Advice rather than a formal guideline (Table 1). In clinical practice, SSLs identified outside the rectum are recommended for endoscopic removal irrespective of size. For rectal lesions, resection is typically advised for SSLs ≥ 5 mm and only SSLs < 5 mm may be selectively observed, though the prevailing tendency is still toward removal. A common exception to universal resection applies to diminutive HPs < 5 mm confined to the rectosigmoid, which are often left in situ because of their negligible or no malignant potential between screening or surveillance interval.

**TABLE 1:** Comparison of endoscopic management and surveillance guidelines for serrated lesions: Western and Japanese guidelines.

Guidelines/Year/Country	≤ 5‐mm SSL	6–9‐mm SSL	10–19‐mm SSL (no dysplasia)	SSLD (any size)	≥ 20‐mm SSL	Surveillance interval
ESGE Polypectomy/2024/Europe	CSP recommended; include 1–2‐mm normal margin	CSP recommended; include 1–2‐mm normal margin	pCSP recommended	Prefer HSP/EMR for en bloc when feasible; avoid cold piecemeal	pCSP/pEMR acceptable	No surveillance recommended for SSL < 10‐mm (screening interval applies)
USMSTF/2020/USA	N.A.	N.A.	EMR/pCSP case‐by‐case	EMR	EMR/pCSP case‐by‐case	SSL < 10 mm: 5 years; SSL ≥ 10 mm or SSLD: 3 year
JSGE Colorectal Polyps GL/2021/Japan	Resection is not indicated	Resection is not indicated	EMR	EMR/ESD per standard indications	N.A.	N.A.
JGES Screening & Surveillance 2021/Japan	Resection is not indicated	Resection is not indicated	EMR	EMR/ESD per standard indications	N.A.	Any SSL or SSLD: 3–5 year

Abbreviations: CSP, cold snare polypectomy; pCSP, piecemeal cold snare polypectomy; HSP, hot snare polypectomy; EMR, endoscopic mucosal resection; pEMR, piecemeal endoscopic mucosal resection; SSL, sessile serrated polyp; SSLD, sessile serrated polyp with dysplasia; N/A, not applicable.

#### 3.1.2 Japanese Perspective

The Japanese approach to the management of serrated lesions is characterized by high‐resolution colonoscopy and JNET and pit classifications for precise, in vivo differentiation of serrated lesion subtypes, particularly between SSL and HP, to guide a more selective resection strategy [15, 17, 21, 34].

The Japan Gastroenterological Endoscopy Society (JGES) Guidelines for colorectal endoscopic submucosal dissection (ESD)/endoscopic mucosal resection (EMR) (2020) allows observation of typical distal hyperplastic polyps ≤ 5 mm but does not describe SSL (SSA/P) resection indications, reflecting limited evidence [35]. The Japanese Society of Gastrenterology (JSGE) Colorectal Polyps Guidelines (2021) classifies SSA/P as a neoplasm and proposes endoscopic resection, noting ≥ 10 mm as a practical threshold, and specifies that SSLD should be resected regardless of its size because of the malignant transformation potential of cytologic dysplasia [36]. The JGES Colonoscopic Screening & Surveillance Guidelines (2020) then explicitly adopts SSL and proposes resection for SSLs ≥ 10 mm or when dysplasia is suspected, grounding the 10‐mm cutoff in a case control study [34]. Lastly, the JGES “Colorectal Cold Polypectomy” guidelines (2021) do not issue a specific indication for SSL because direct evidence remained limited [37] (Table 1).

### 3.2 Selection of Endoscopic Resection Technique for SSL: Western Versus Japanese Perspectives

#### 3.2.1 SSL

##### 3.2.1.1 Western Perspective

**FIGURE 2:**
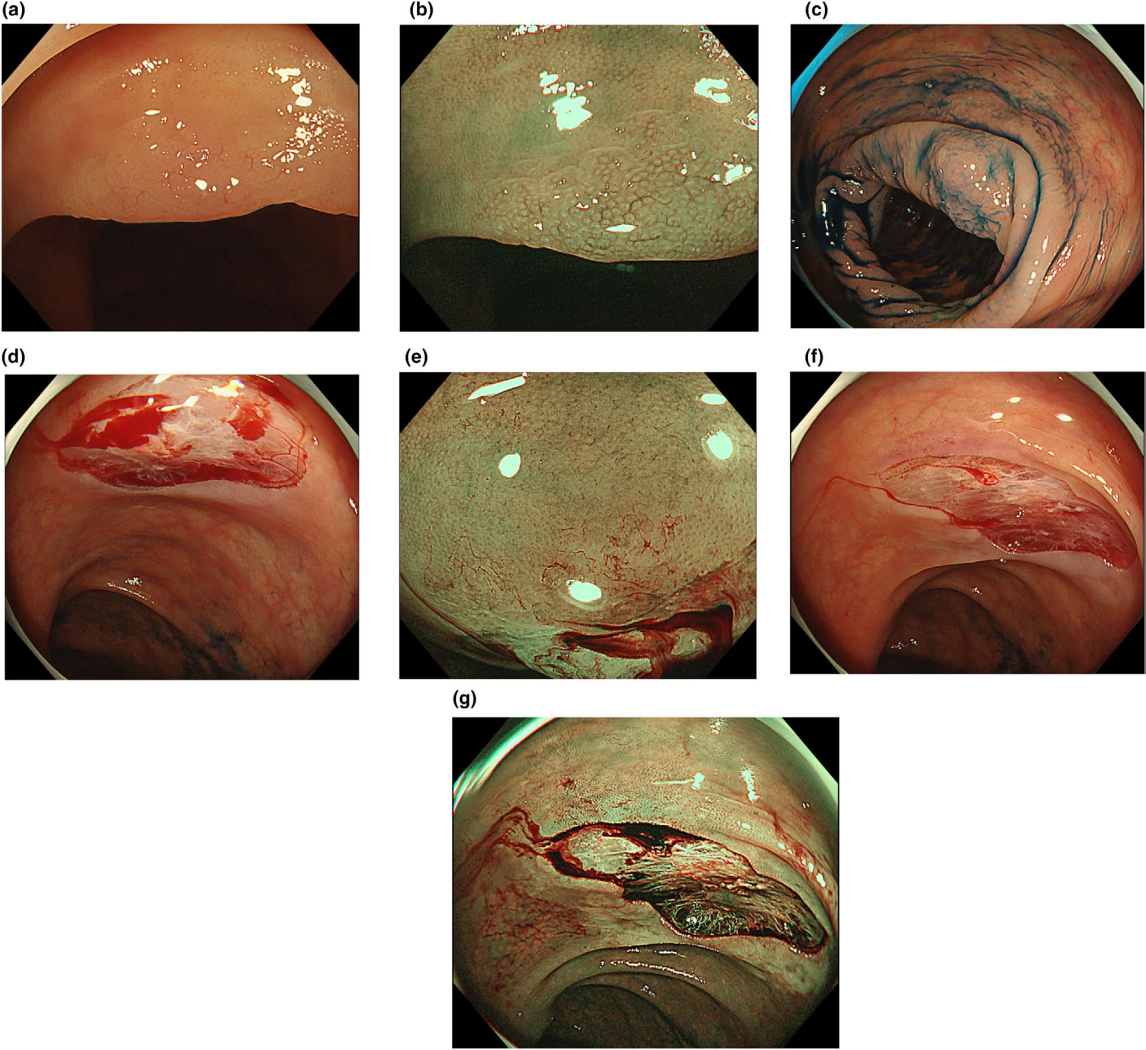
Piecemeal cold snare polypectomy (CSP) of a 15‐mm sessile serrated lesion (SSL) in the ascending colon. (a) The lesion is partially obscured, with approximately half of its circumference extending behind a fold. (b) On NBI, the lesion corresponds to Japan NBI Expert Team (JNET) type 1, showing dilated vessels and dark spots inside crypts. (c) Chromoendoscopy with indigo carmine dye spray showed no endoscopic findings suggestive of dysplasia. (d) The first CSP session. (e) A tiny residual lesion was detected on the anal side on magnified NBI. (f) The second session achieved complete removal of the residual lesion. (g) NBI confirmed no residual lesion.

Western guidelines endorse cold snare polypectomy (CSP) as the standard technique for most non‐complex serrated lesions [32, 33]. For diminutive and small serrated lesions, this recommended technique is routinely performed due to its higher rate of en bloc resection and very low incidence of adverse events. For 10–19‐mm SSL without dysplasia, piecemeal CSP, with an effort to incorporate a 1–2‐mm rim of surrounding normal mucosa, is recommended [32] (Figure 2). This helps to achieve complete resection, reduce risk of residual lesion and recurrence with very low severe adverse events when meticulous lesion with margin assessment and high‐quality safe technique are applied [31, 38]. For ≥ 20‐mm lesions, cold piecemeal EMR demonstrates favorable safety versus hot EMR; efficacy appears better for SSL than for adenomas [39, 40, 41, 42], supporting a cold‐first approach in selected SSLs, mostly in the right‐side colon. Also, some authors report an optional submucosal injection can be used for cold EMR.(Table 2) However, in practice, cold or hot snare piecemeal polypectomy or EMR is selected according to the endoscopist's preference and expertise.

**TABLE 2:** Outcomes of cold snare polypectomy with/without submucosal injection for sessile serrated lesions (≥ 10 mm) reported in high‐volume series (≥ 100 lesions).

Author (year)/Country	Target lesions	Mean size (mm)	Technique	Present of SSLD	Local recurrence rate (median follow‐up)	Major adverse events
van Hattem WA (2021)/Australia	SSLs ≥ 20 mm	25.0	CSP	N.A.	4.3% (6 months)	0%
Yoshida N (2021)/Japan	10‐19 mm SSLs	11.8	CSP	N.A.	5.0% (18 months)	0%
Barros RA (2021)/Argentina	(a) 10‐19 mm SSLs (b) SSLs ≥ 20 mm	14	CSP	N.A.	(a) 6.0%, (b) 10.1% (6 months)	0%
Kimoto Y (2022)/Japan	SSLs ≥ 10 mm	14 (median)	CSP	N.A.	0% (7 months)	0%
Tutticci NJ. (2015)/USA	SSPs ≥ 10 mm in the proximal colon	17.5	Cold EMR	1.2%	0.95% (Mean 5 months)	0%
Mangira D (2020)/Australia	SSLs ≥ 20 mm	25.5	Cold EMR	N.A.	5.5% (5 months)	3.8% clinically significant post‐EMR bleeding
McWhinney CD (2021)/USA	SSLs ≥ 10 mm	17.2	Cold EMR	3%	8.0% (12.4 months)	0%
Williams TJ (2025)/Australia	(a) 10‐19‐mm SSLs (b) SSLs ≥ 20 mm	20	Cold EMR	0%	(a) 1.4%, (b) 4.1% (6 months)	0%

Abbreviations: CSP, cold snare polypectomy; EMR, endoscopic mucosal resection; SSL, sessile serrated polyp; SSLD, sessile serrated polyp with dysplasia; N/A, not applicable.

##### 3.2.1.2 Japanese Perspective

In contrast, the Japanese guidelines restrict CSP to adenomas < 10 mm and do not recommend it for SSLs, particularly 10–19 mm, because direct, Japan‐specific evidence remains limited. Within this framework, SSLs ≥ 10 mm are generally treated with EMR.

#### 3.2.2 SSL with Cytologic Dysplasia

##### 3.2.2.1 Western Perspective

Because SSLD carries a higher risk of advanced histology and covert malignancy, Western guidelines recommend definite endoscopic resection [32, 33]. En bloc resection is preferred whenever feasible to ensure clear margins and facilitate accurate histopathology. For 10–19‐mm lesions without endoscopic signs of deep invasion, cold or hot snare polypectomy with consideration of submucosal injection is recommended; and many centers perform adjunctive margin treatment with thermal ablation to reduce recurrence. Piecemeal CSP techniques are generally not recommended for SSLD because it precludes margin assessment and may increase risk of residual neoplasia and local recurrence. For ≥ 20‐mm lesions, piecemeal hot EMR is widely used, whereas ESD is considered in expert hands when en bloc resection is desired for lesions with surface depression, non‐lifting sign or if deep invasion is suspected.

##### 3.2.2.2 Japanese Perspective

Current Japanese guidelines recommend resection for SSLs ≥ 10 mm or when dysplasia is suspected, but they do not specify size‐based technique selection for SSLD and do not explicitly mandate en bloc resection for them. Therefore, technique selection is at the discretion of the endoscopist and expertise, and lesion characteristics, with emphasis on achieving clear margins and consistent reliable histopathology assessment [34, 35, 36, 37].

## 4 Challenges and Future Directions

As we have outlined the current landscape, and the difference in approach between Japanese and Western practice, the next—and arguably most important—step is how best to integrate these insights into practical universal strategies that reduce post‐colonoscopy and post‐imaging colorectal cancer (PCCRC) while maintaining a high‐quality, safe and cost‐effective approach in clinical practice. In this context, we highlight three tightly linked themes: (i) authors' perspective on indications and technique choice; (ii) ongoing prospective studies that will refine risk stratification; (iii) surveillance and quality improvement strategies.

### 4.1 Authors' Perspective on Indications and Technique Choice

The Japanese clinical guidelines for serrated lesions were last revised approximately 5 years ago, and substantial additional evidence has accumulated since then. Based on this evolving evidence and our clinical experience, we advocate proactive resection at the time any SSL is suspected with high confidence, regardless of its size or location, provided that the lesion can be safely and completely removed. This advocacy is impacted by several considerations: (i) dysplasia has been reported even in SSLs < 10 mm, indicating that lesion size alone does not reliably exclude advanced histology [43]; (ii) endoscopic predictive probability for diagnostic accuracy of SSLs—particularly sensitivity—remains suboptimal across modalities; (iii) long‐term, periodic endoscopic surveillance is not acceptable to all patients especially for low‐risk lesions and may be inconsistently implemented; (iv) the increasing adoption of CSP, which is associated with a low rate of procedure‐related adverse events, [44, 45] and lowers the threshold for safe removal of small serrated lesions.

For SSLs without dysplasia, we consider CSP reasonable for lesions up to 10 mm in carefully selected cases, if it is performed en bloc with appropriate incorporation of a 1–2 mm surrounding normal mucosal margin, adequate specimen retrieval, and clear documentation of margins [46, 47]. For 10–19‐mm SSLs, we currently favor conventional EMR until higher‐quality evidence directly applicable to Japanese practice becomes available; “cold EMR” in this size range may be regarded as investigational and center‐dependent. For lesions ≥ 20 mm, hot EMR preferably en bloc or ESD should be selected based on lesion morphology, suspected invasion, and local expertise. Right‐sided ESD should be limited to expert centers with demonstrated high en bloc, low perforation, and adverse event rates.

**FIGURE 3:**
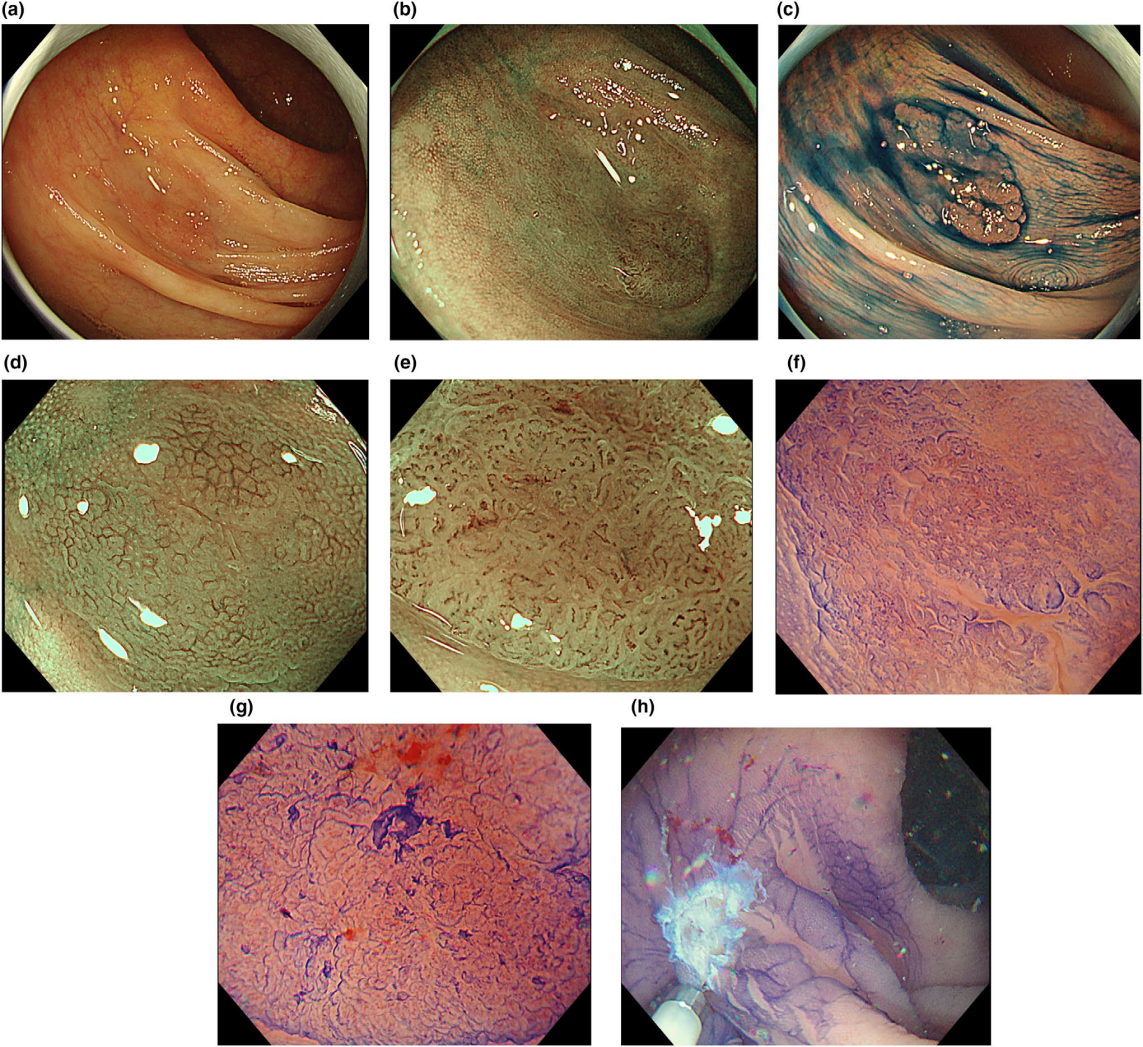
Underwater endoscopic mucosal resection (UEMR) of a 15‐mm SSL with dysplasia in the ascending colon. (a) White‐light imaging showed a focal area of slight redness with an avascular mucosal pattern. (b) NBI revealed an 8‐mm brownish area. (c) Indigo carmine dye spray clearly delineated the lesion and its border. (d) Magnified NBI showed dark spots inside crypts on the left side of the lesion surface, consistent with JNET Type 1. (e) The right side of the lesion surface showed JNET Type 2B. (f) Type II open pit pattern was observed on the left side of the lesion surface. (g) Type V_I_ (low‐grade) pit pattern was observed on the right side of the lesion surface. (h) En bloc resection was achieved by UEMR.

For SSLD, we prefer an en bloc strategy whenever technically feasible. In our view, en bloc resection for SSLD is desirable to facilitate accurate histopathologic diagnosis, clarify margins, and reduce local recurrence risk. When JNET or pit‐pattern findings suggest high‐grade dysplasia/mucosal cancer or submucosal invasion, en bloc removal by hot EMR (Figure 3) or ESD—based on lesion morphology and the discretion of the endoscopist and available expertise—is recommended. If the dysplastic component appears limited and consistent with low‐grade adenoma, piecemeal resection may be acceptable provided that the dysplastic area itself is removed en bloc, with careful margin documentation, incorporation of an appropriate rim of surrounding normal mucosa, and complete retrieval of all fragments.

### 4.2 Ongoing Prospective Studies

The proportion of dysplasia and cancer coexisting within large SSL is a key determinant of optimal management for SSL and SSLD, yet this issue has not been adequately investigated. To address this gap, we have initiated a prospective cohort focusing on large SSLs (UMIN000056765; ONE PIECE study), to hopefully better define the prevalence of dysplasia and early cancer in large SSLs and clarify which endoscopic morphologic features predict advanced histology.

Similarly, to evaluate the role of CSP resection techniques for intermediate‐sized SSLs in a Japanese context, we are conducting a randomized controlled trial for 10–20‐mm SSLs (jRCT1062230020; CONCISE trial). This study compares CSP‐based strategies with conventional hot EMR, with particular attention to completeness of resection, recurrence, and safety. Together, these studies aim to provide the evidence‐based information required to refine size and risk acceptable indications for cold versus hot snare resection techniques, and to guide future updates of Japanese and international guidelines.

#### 4.2.1 Surveillance and Quality Improvement Strategies

##### 4.2.1.1 Surveillance

We intentionally avoided reiterating PCCRC causes here [9, 48, 49]. Instead, we focus on practical strategies that mitigate risk: (i) interval selection guided by histology and completeness of resection; (ii) early endoscopic reassessment of the resection site after piecemeal resection or when margins are indeterminate; (iii) continuous quality metrics and consistent high‐quality documentation bundles (optimized bowel preparation and mucus clearance, IEE such as NBI, BLI and linked color imaging (LCI) use, and post‐resection site imaging). These measures might allow safe interval extension in low‐risk, well‐documented cases and appropriate shortened interval for individuals with lesions adequately documented and considered low risk, and when overall quality is suboptimal.

Western guidance emphasizes lesion size and number‐stratified intervals with a firm 3‐year anchor for SSL ≥ 10 mm or any SSLD/TSA, and advises early endoscopic assessment after piecemeal resection or indeterminate margin status [4, 50, 51]. ESGE strongly recommends that patients with any serrated polyp < 10 mm without dysplasia do not require endoscopic surveillance and should be returned to average risk screening or other findings‐based surveillance intervals [51]. In contrast, Japanese guidance is dysplasia‐centered and more conservative regarding universal “cold‐first” adoption: which after complete resection, follow‐up is typically 3–5 years, with 6–12‐month shortened index interval surveillance of the post‐resection scar after any piecemeal or indeterminate‐margin resections [34], and with a stronger emphasis on definitive histopathology.

As evidence remains limited and variable, we're of the strong opinion that surveillance recommendations should be guided first by histology and completeness of resection; followed by examination and documentation quality, with piecemeal resection requiring an earlier surveillance of post‐resection scar. Our recommended intervals are as follows:

- SSL ≥ 10 mm or any serrated lesion with dysplasia (SSLD/TSA) → 3 years.
- 1–2 SSL < 10 mm, complete resection → 5–10 years.
- ≥3 SSL < 10 mm → 3 years.
- Serrated polyposis syndrome (SPS) → 1 year.
- Piecemeal ≥ 20 mm or indeterminate margins → 6–12 months, then resume the interval by risk tier as above.

##### 4.2.1.2 Optimizing and Improving the Detection of Serrated Lesions

In addition, optimizing and improving the detectability of serrated lesions is necessary to enhance the quality of colonoscopy [52, 53]. Techniques include consistent high‐quality colonoscopy with IEE‐first approach, particularly in the proximal colon where SSLs are often flat, pale with indistinct margins, and performing right colon retroflexed view [54] or second look forward view should be emphasized. Contemporary randomized data support considering IEE as the default observation mode in such settings, with targeted dye spray when borders remain indistinct [55, 56, 57, 58]. Large parallel RCTs have shown higher overall and serrated detection with LCI versus high‐definition white light [55]; tandem trials indicate that BLI/NBI can improve proximal detection, with BLI lowering proximal adenoma miss rates [57]. A recent multicenter randomized Japanese study also suggests that a second‐pass acetic acid–indigo carmine mixture (AIM) increases proximal serrated lesion detection without major adverse events [58]. Recent advances in artificial intelligence have also demonstrated potential to improve both detection and characterization of colorectal lesions, including sessile serrated lesions [59, 60]. Pragmatically, programs should standardize for optimized bowel preparation ensuring adequate mucus clearance, use IEE, consider AIM or acetic acid chromoscopy [61] for proximal, mucus‐capped, or poorly demarcated lesions when appropriate, and require photo‐documentation of resection base to justify interval extension—or, when quality is suboptimal, interval shortening.

## 5 Conclusions

Although high‐quality evidence for management of serrated lesions with and after endoscopic resection remains limited, the direction toward optimized evidence‐based guidance is clear and evolving. Continued clarification of serrated lesion biology, together with adopting a disciplined approach to improve detection by including use of IEE and chromoscopy, judicious selection of CSP, EMR, or ESD, and risk‐aligned surveillance with early shortened index surveillance after piecemeal resection or advanced histology with indeterminate margins—provides a practical care plan to reduce both the incidence and mortality of CRC and PCCRC. By harmonizing Japanese and Western strengths, definitive histopathology assessment and safety on one hand, ease of adoption and continuous quality metrics on the other, it is possible to deliver more effective care with improved safety while ongoing prospective studies provide crucial data to guide consensus universal, safe and appropriate intervals, and responsibly expanding indications for cold resection techniques.

## Author Contributions

T.U. conceived the study and led the project, drafted the manuscript (writing – original draft), and integrated input from all co‐authors. Y.Y., K.I., Y.T., and H.R. provided critical intellectual input and important advice and contributed to manuscript revision (writing – review and editing). H.R. reviewed and edited the manuscript for English language as a native English speaker. H.T. and K.S. assisted with manuscript writing and revisions. Y.I., H.H., and S.K. contributed to manuscript review and editing. All authors reviewed and approved the final manuscript.

## Funding

The authors have nothing to report.

## Conflicts of Interest

Toshio Uraoka, Yasushi Yamasaki, Kenichiro Imai, and Yoji Takeuchi have received lecture fees from Olympus Co. Yasushi Yamasaki and Kenichiro Imai are Associate Editor of Digestive Endoscopy. The other authors declare no conflicts of interest for this article.
